# Ceramics, Glass and Glass-Ceramics for Personal Radiation Detectors

**DOI:** 10.3390/ma14205987

**Published:** 2021-10-12

**Authors:** Szymon Świontek, Marcin Środa, Wojciech Gieszczyk

**Affiliations:** 1 Faculty of Materials Science and Ceramics, AGH University of Science and Technology, al. Mickiewicza 30, 30-059 Kraków, Poland; msroda@agh.edu.pl; 2 The Henryk Niewodniczański Institute of Nuclear Physics, Polish Academy of Sciences, ul. Radzikowskiego 152, 31-342 Kraków, Poland; wojciech.gieszczyk@ifj.edu.pl

**Keywords:** thermoluminescence (TL), high energy dose dosimetry, ionizing radiation, brachytherapy, rare-earth elements

## Abstract

Different types of ceramics and glass have been extensively investigated due to their application in brachytherapy, radiotherapy, nuclear medicine diagnosis, radioisotope power systems, radiation processing of food, geological and archaeological dating methods. This review collects the newest experimental results on the thermoluminescent (TL) properties of crystalline and glassy materials. The comparison of the physico-chemical properties shows that glassy materials could be a promising alternative for dosimetry purposes. Furthermore, the controlled process of crystallization can enhance the thermoluminescent properties of glasses. On the other hand, the article presents information on the ranges of the linear response to the dose of ionizing radiation and on the temperature positions of the thermoluminescent peaks depending on the doping concentration with rare-earth elements for crystalline and glassy materials. Additionally, the stability of dosimetric information storage (fading) and the optimal concentration of admixtures that cause the highest thermoluminescent response for a given type of the material are characterized. The influence of modifiers addition, i.e., rare-earth elements on the spectral properties of borate and phosphate glasses is described.

## 1. Introduction

Thermoluminescent dosimetry is a science which deals with measuring doses of ionizing radiation with the use of TL detectors. Thermoluminescence (TL) dosimetric studies have significant impact on many fields useful in ordinary life such as: radiotherapy [1], nuclear medicine diagnosis [2], radioisotope power systems [3], radiation processing of food [4], geological and archaeological dating methods [5,6], radiation shielding materials [7,8]. Due to the increasing use of ionizing radiation in technology, it seems that we must search for better materials suitable for radiation shielding. The amorphous structure of the glass, which is characterized by exceptional chemical resistance, seems to be ideal for this type of application. Moreover, the thermoluminescent materials are used in measurements of terrestrial and cosmic radiation [9] or radionuclide sources [10]. The phenomenon of thermoluminescence depends on the light emission as a result of heating a sample that was previously exposed to ionizing radiation. The luminescence mechanism in this case can be considered as phosphorescence caused by increasing temperature. The light emission can be observed directly during the first heating process. Luminescence can also be observed again after radiation exposure of a specific wavelength. The phenomenon of thermoluminescence occurs mainly in dielectrics [11] but also in organic materials [12]. The thermoluminescence effect was firstly observed and described in 1663 by the English physicist Robert Boyle [13]. Thermoluminescence was acknowledged in a diamond which had been heated to the temperature of the human body. Then, in 1904 Maria Skłodowska-Curie observed temperature-stimulated luminescence in fluorite (CaF_2_) [14]. She noticed the relationship between the exposure of natural calcium fluoride to radium radiation and the intensity of its light emission. The practical application of the thermoluminescence phenomenon in dosimetry was not developed until the end of the first half of the twentieth century, when the Randall-Wilkins theory was formulated [15]. Since the discovery of the phenomenon of thermoluminescence, many monographs and review articles containing the physical basis of this phenomenon have been published. The books “Thermoluminescence and Thermoluminescent Dosimetry”, by Yigal S. Horowitz [16] and “Thermoluminescence of solids” by S.W.S. McKeever [17], are worth mentioning. In addition, many summary reviews have been written on this subject, such as: “Thermoluminescence dosimetry and its applications in medicine” by T. Kron [18], “Thermoluminescence and its Applications: A Review” by K.V.R. Murthy [19] or “Versatility of thermoluminescence materials and radiation dosimetry—A review” by A. Duragkar [20]. The purpose of the following review article is to summarize the knowledge of the thermoluminescent properties of glassy and glass-crystalline materials.

The vast majority of the analytical methods applied in the glow-curve analysis are based on one recombination and one trapping level. Unfortunately, there is no ideal TL material which can be thoroughly specified by this model. Given the degree of defect centers and arrangement complexity in glassy networks, it is an extremely difficult case to understand the physical principles of thermoluminescent phenomena for amorphous systems. Therefore, the calculated values of activation energy *E_A_* and frequency factors f are, in fact, effective values. The glow curve shape depends on several factors, such as: the TL material composition, kind of dopants, types of defect centers induced by irradiation, the type of ionizing radiation and its dose level. The shape of the TL glow-curve for glassy samples might be explained by the presence of multiple trapping points which increase the probability of re-trapping mechanism. It is widely understood that the shape factor of a standard first order peak is about equal to 0.42 [21]. Simultaneously, when the value of shape factor is greater than 0.52, we are dealing with second order kinetics mechanism, respectively. Moreover, it has been demonstrated that the various heating rates can affect on TL glow-curve shape along with different values of trapping parameters [22]. In general, with the increasing heating rate, the TL peak temperature shifts to higher temperatures region. The increase in the intensity of thermoluminescence signal with respect to a radiation dose is caused by increasing number of excited electrons. The standard shape and position of glow TL peaks can be changed in respect to location of trapping levels in the glassy network [23]. Primarily, when the shape of the glow curve is asymmetric, wider at the low temperature region than at the high temperature region, then the kinetic process of thermoluminescence is attributed to the first order [24]. It can be equivocally expressed as no re-trapping luminescence process during which all the charge carriers freed from the trap centers follow directly to the recombination points. The thermally stimulated luminescence (TL) can be used to determine the optic properties of the glasses under study with radiation doses [25].

The process of irradiation might induce the displacement of atoms or electron defects which include the changes in the valence state of network atoms, modifier, or impurity atoms. Moreover, the changes in the glow curve shape can be used to deduce the damage created by the high radiation to the thermoluminescent material [26]. The extent of disordering in glass network can be deduced from Urbach energy (Urbach tail) which described the localized states present in the bandgap [27]. During irradiation, atoms are ionized as the bound electrons interact with high energy particles. This interaction leads to the generation of electron-hole pairs which got trapped at structure defects or glass impurities having energy states within the forbidden band. As a result of this ionization process, the electron-hole pairs in the glass structure are generated after exposing to ionizing radiation. In the case of borate and phosphate glasses, radiation defects are related mainly to the absence or excess of oxygen atoms in the amorphous glass structure [28,29,30]. For silicate, telluride or heavy metal fluoride glasses, radiation defects may be also color centers or structural vacancies [31,32]. Therefore, when heat or light stimulate the structure of the glass, the recombination of charges results in luminescence.

## 2. Materials and Methods

The thermoluminescent detector was used in ionizing radiation dosimetry for the first time during nuclear testing as part of the Manhattan Project in the United States since 1942 [33]. Nowadays, we are observing a tremendous progress in the thermoluminescence dosimetry from that moment [34,35,36,37]. The most commonly used material in TL detectors is crystalline lithium fluoride. There are a several types of detectors based on this compound: MCP detector (LiF doped Mg, Cu and P), MTS detector (LiF doped Mg and Ti) or MTT detector (LiF doped Mg and Ti with ten times more titanium and almost three times less magnesium in comparison with the MTS detector) [38]. The most important advantages of these detectors are high sensitivity and a cross-section for interaction with ionizing radiation which are similar to that of human tissues. Natural lithium contains 92.5% of the ^7^Li isotope and 7.5% of the ^6^Li isotope. The TL detectors also use lithium depleted or enriched in the ^6^Li isotope, which corresponds to three subtypes: MCP-N (natural abundance of lithium isotopes), MCP-6 (^6^Li-enriched) and MCP-7 (^7^Li-enriched) [39]. MTS-N detectors are characterized by a linear thermoluminescence response to approx. 1 Gy, and the reduction in the signal at room temperature is estimated at several percent per year. Moreover, they are characterized by good tissue similarity, therefore they are used in environmental and individual dosimetry. The MCP-N detector is characterized by thirty times greater sensitivity than the MTS-N detector and three times lower own background. This allows the measurement of radiation doses at the level of 200 nGy, which is a result 100 times lower in comparison with the MTS-N detector. The response of the MCP-N detector is linear up to a few Gy and has good stability over time. The spontaneous decrease in the stored radiation signal over time amounts to approx. 5% per year in the room temperature. The MTT-N detector is more efficient than the MTS-N detector, but its sensitivity is approximately 2.5 times lower. Therefore, the minimum measured dose is 1 mGy.

**Figure 1:**
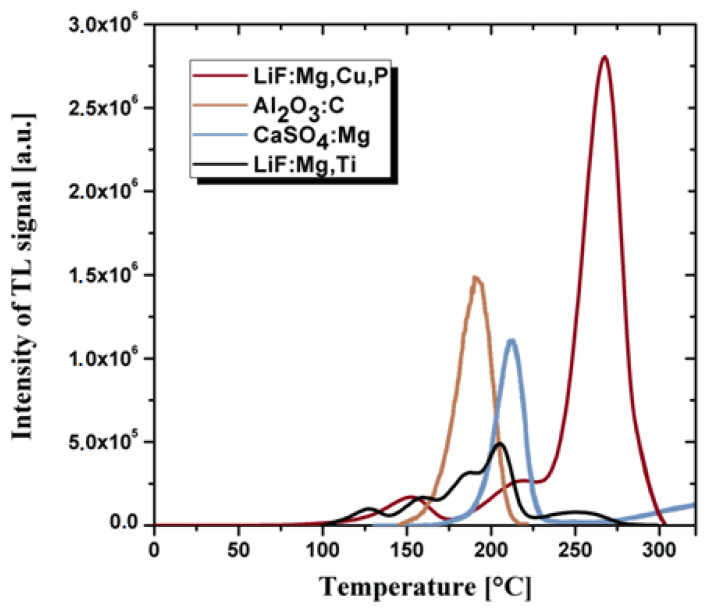
The comparison of the thermoluminescence glow curves for various crystalline materials used commercially as TL detectors after 10 mGy radiation dose. Data collected from the publications [38,41,42].

Fluorides and calcium sulphates doped Tm or Dy and Al_2_O_3_ doped carbon are also successfully used as thermoluminescent materials [40,41]. However, in practice, they are less often used items than crystalline doped LiF due to the different cross-section for interaction with radiation in comparison with human tissues. The most important technical parameters that prove the quality of thermoluminescent materials include: a wide range of energy of the measured doses, the linearity of the response to the radiation dose, potentially small geometric dimensions, the lack of need for power supply and chemical resistance to environmental factors. An undesirable feature of the thermoluminescent material is the high annealing temperature during the dose reading procedure. Thermoluminescent detectors are usually in the form of sintered bulk ceramics or powders. The most commonly used TL detectors are based on the doped: LiF, Al_2_O_3_, CaF_2_ and CaSO_4_ [40,41,42]. The comparison of the thermoluminescence glow curves for the same radiation dose for the crystalline TL materials is presented in the Figure 1.

The glass matrix with optically active dopants may also be of interest for developing dosimetric detectors. The borate glasses have a high transparency and high solubility for the rare-earth ions among other oxide glasses, i.e., silicates, phosphates and tellurite glasses [43]. However, due to their multiple phases, high phonon energy and low moisture resistance, borate glasses have some limitations to their optical gain medium. On the other hand, the borosilicate glasses have a better thermal stability, higher value of refractive index and lower dispersion as well as higher chemical resistance [44]. Therefore, these glasses are widely used for optoelectronics [45] and optical lenses applications [46] and also as glass cookware due to low thermal expansion coefficient [47]. The incorporation of rare-earth elements to the glass matrix can induce thermoluminescence effect and produce optical active materials [48]. These dopants act as luminescent centers what enables radiation processes.

The incorporation of alkali ions in the borate network can reduce the risk of different phases formation in the glass by converting BO_3_ triangle borate units to BO_4_ tetrahedra [49]. The addition of fluoride ions to oxide glasses induces the special oxyfluoride properties which combine the low phonon energy and value of refractive index of fluorides with thermal stability towards devitrification of the oxide glasses. For example, after incorporation of lithium ions to borate glass network, the negative charge of the boron-oxygen tetrahedron is compensated by lithium cations [50]. Moreover, alkali fluoride (i.e., LiF, KF or NaF) can be introduced to borate network up to 45 mol%. The rearrangement of lithium ions due to its small radius can likewise be caused by irradiation or glass matrix imperfection. Therefore, it enables formation of negative point defects which play a role of hole traps. On the other hand, oxygen vacancy formed a positive point defect which play a role of electron traps. That explains why, after lithium oxide addition to borate matrix, boron-oxygen triangle attaches an additional oxygen atom forming boron-oxygen tetrahedron. The oxyfluoride glasses from the LiF-B_2_O_3_-SiO_2_ system are characterized by favorable thermoluminescent properties [51]. The increase in lithium fluoride contribution from 20 to 40 mol% in the borosilicate glass causes efficiency enhancement of the thermoluminescence signal. Moreover, the process of controlled crystallization based on DSC curves can increase TL sensitivity, narrowing and shifting the main TL peak to the lower temperatures. The glass-ceramics with 40 mol% LiF containing LiBF_4_ optically active phase exhibits similar level of TL signal to commercially used doped LiF materials [52].

The addition of a heavy metal oxide to the borate network increases the transmission of light in the mid-infrared region and decreases the melting temperature and the phonon energy [53]. Alkali-heavy metal oxide borate glasses show high UV–vis–NIR transparency, low melting and glass transition temperatures, low phonon energy, low thermal expansion coefficient, good mechanical strength and chemical stability [54]. Among various glass hosts, a borate matrix is a proven good radiation absorber with a cheap processing [55]. The effective atomic number Z_eff_ of network former B_2_O_3_ is about 7.35 which is close to that of human tissue (Z_eff_ = 7.42) [56]. Heavy metal oxides, such as BaO, decrease the phonon energy of the glass matrix, and simultaneously increase the efficiency of luminescence [57]. It has been observed that the addition of BaO up to 25 mol% to borate glass could help improve the thermoluminescence intensity.

In some cases, commercial borate glasses exhibit UV charge transfer absorption because of the trace iron impurities presence (largely Fe^3+^ ions) [58]. It was proved that transition metal (TM) ions even for the ppm level in glasses could produce strong UV absorption. Moreover, the charge transfer mechanism for glasses has been thoroughly classified based on trace transition metal impurities content (such as Fe^3+^ or Cr^6+^) [59]. The increased absorption in the range of ultraviolet results from an electron transfer mechanism (electron transition from the orbital of a coordinating oxygen atom to an orbital of the metal ion). Abdelghany and ElBatal have observed charge transfer absorption bands in the UV range for a various undoped borate glasses [60,61]. Therefore, the trace iron impurities (largely Fe^3+^ ions) which are present in the chemical reagents/raw materials can induce UV absorption mechanisms in different glass systems.

The first thermoluminescent material, based on lithium borate polycrystal Li_2_B_4_O_7_ doped with manganese, was introduced in radiation dosimetry by J.H. Schulman in 1967 [62]. Unfortunately, the TL sensitivity of Li_2_B_4_O_7_:Mn was quite low and it was impossible to use this material for commercial purposes. Moreover, the reproducibility and the linearity of its dose-response for high energies were insufficient for strict environmental requirements. Subsequently, M. Takenaga significantly improved the TL sensitivity of lithium borate phosphor by using Cu dopants instead of Mn ions [63]. Nowadays, a high efficiency of fluorescence was obtained for borate glasses doped with europium ions [64]. The significant improvements of dosimetric features can be modified by using co-dopants such as transition metals [65]. However, to our present knowledge, the correct mechanism of the TL phenomenon for doped lithium tetraborate glasses is still under discussion. On the other hand, polycrystalline probes of lithium tetraborate exhibit two well-separated thermoluminescent peaks which are related to two different types of trapping points [66]. Electrons can be trapped by oxygen vacancies while holes are trapped by lithium vacancies in Li_2_B_4_O_7_ matrix. The monotonous decrease in luminescence signal intensity with higher temperature is due to gradual release of trapped charge carriers. By the thermal energy absorption process, the trapped holes in borate network are freed and can combine with electrons released from the oxygen vacancies which results in emission of the light. The symmetry factors f for lithium tetraborate glasses irradiated with various radiation dose and measured with different heating rates are greater than 0.52, which suggests the second order kinetic of luminescence process [64]. It is widely known that higher order of luminescence kinetic means greater probability of re-trapping processes due to many trapping points in glassy matrix [67]. The main type of radiation damages in lithium borate glasses are Frenkel pairs in the form of vacancies of oxygen and boron with inter-nodal ions [68]. However, the thermal stability of the defect points produced by ionizing radiation has always been a topic of discussion. Unfortunately, electron and hole traps for lithium tetraborate glasses exhibit weak thermal stability with respect to ionizing radiation [69].

The phosphate glass dosimeters are currently of particular interest due to their high thermoluminescence (TL) yield and the simplicity of fabrication and modification for various applications in terms of their chemical and physical properties [70]. Phosphate glasses are often used as biomaterials because they are often similar in chemical composition to the natural bone [71]. However, pure phosphate glasses are often not chemically stable for practical applications [72]; they are characterized by low melting temperature, small value of refractive index and uniquely high thermal expansion coefficient when compared to silicate glasses. Therefore, they can be used as high-power lasers, sealing materials, glasses for medical uses and ionizing radiation dosimetric purposes. A typical network of phosphate glasses contains a polymeric structure dominated by linkages between the PO_4_ tetrahedra. For vitreous P_2_O_5_, these groups are connected to adjacent units by three of their four vertices, while one place is occupied by a terminal double-bounded oxygen atom. The extent of glass formation in alkali and alkaline earth binary phosphates is generally larger than in silicates and the borates [73,74]. It is the responsibility of the phosphate rings by the modifier ions to form phosphate chains with varying chain lengths [75].

P_2_O_5_ is often used in the optically active glasses due to the shift of the absorption cut-off wavelength to the near infrared region (NIR) [76,77]. The advantage of phosphate glass is its higher solubility of rare earths in its structure in comparison with silicate glasses. Moreover, the lithium fluoride added to phosphate glass causes a decrease in thermal fading at the room temperature [78]. Rare-earth ions, in their trivalent state, act as fluorescence activators when doped in the phosphate glass matrix and enhance TL emission. However, photo and thermoluminescence properties of the RE ions are sensitive to various solid-state hosts. They exhibit absorption bands in the near-UV to blue region and sharp emission bands in the entire visible region of the electromagnetic spectrum, which is expected for visible solid-state lasers and light emitting diodes [79]. The amorphous nature of the glasses makes them susceptible to accommodate radiation-induced defects what is also their advantage.

Pure phosphate glasses produce no TL signals [80]. However, lithium-doped phosphate glasses characterize a broad TL peak at the rage of 200–300 °C [81]. On the other hand, barium-doped phosphate glasses feature at least two TL peaks, approximately at 220 and 390 °C, which are attributed to Ba^2+^ ions [82]. The observed TL peaks result from various defects generated by the modifier ions (Ba^2+^ and Li^+^) inserted into the glass matrix. The low temperature peak characterizes an average intensity loss of 57% after 2 days of storage and a reduction to 9% of the initial signal after 4 months of storage. Thus, thermal fading makes using this peak for dosimetry purposes difficult. The high temperature peak also fades in the short term, but it eventually stabilizes at 75% of the initial value [83]. They also give a fast-decaying OSL signal correlated with the low temperature of TL peak. The optically stimulated luminescence signal increases linearly with the received dose for phosphate glasses containing both barium and lithium ions [84]. On the other hand, a 100-h delay readout after irradiation shows the signal decrease to less than 50% of the initial dose value. The content of barium ions Ba^2+^ allows linear characteristics of the dose response in phosphate glasses up to 100 Gy. Moreover, phosphate glass doped with barium oxide is at least one order of magnitude more sensitive to radiation than the glass doped with lithium oxides [85].

The article presents the thermoluminescent properties of mainly borate and phosphate glasses due to the similar cross-section on the interaction with ionizing radiation to human tissues [86,87]. Unfortunately, silicate and telluride glasses have Z_eff_ significantly different than most human organs [88,89]. For this reason, the use of these materials in personal dosimetry is very difficult.

**Figure 2:**
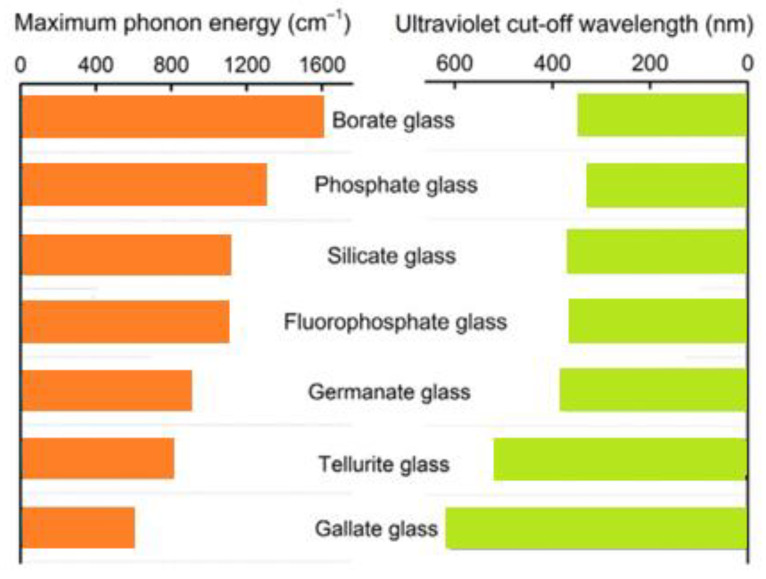
The parallel of highest phonon energy and ultraviolet cut-off wavelength in different glass systems.

Another group are fluoride glasses, the development of which was initiated by the discovery of the glass-forming properties of zirconium fluoride—ZrF_4_. These glasses are based on heavy metal fluoride glasses (HMFG) and are characterized by—compared to oxide glasses—low attenuation of signals in the infrared range (up to approx. 3 μm). At the same time, fluoride glasses readily accept rare-earth elements into their structure, which makes it possible to use them as optical amplifiers in telecommunications bands operating in the near infrared. Figure 2 shows the values of the highest phonon energy together with ultraviolet cut-off wavelength in various glass systems.

## 3. Discussion of the Results

Rare-earth (RE) doped glasses are characterized by promising physical and chemical properties, inclduing: higher thermal stability [90], greater chemical durability [91], superior hardness and elastic modulus than traditional glass network modified by cationic dopants [92]. In general, the rare-earth elements are considered as network modifiers of the glass structure [93,94]. For the phosphate glasses, they are incorporated in the voids between [PO_4_] tetrahedra [95]. The cationic field strength changes with atomic number due to continuous decrease in the ionic radii for the lanthanide elements [96]. Therefore, this phenomenon has a significant effect on the physico-chemical properties of rare-earth doped phosphate glasses.

The thermoluminescence characteristics of various rare-earth-doped glasses for high-dose gamma dosimetry purposes have been studied in recent years [97,98,99,100,101,102,103,104,105,106,107,108,109,110,111]. Scientific research has mainly been focused on extending the saturation limit of dopants in glass matrixes and improving their dosimetry features by varying their composition and concentration.

A thermoluminescent detector should have a wide linear relationship between the integrated TL intensity and the received radiation dose [112]. It enables greater potential of the material for various applications. Many researchers have noticed an enhancement in the TL intensity of glasses after the addition of rare-earth ions to the glass. The ranges of the linear thermoluminescent dose response for selected glasses doped with rare-earth elements are presented in Table 1. The rare-earth ions usually convey a significant amount of the absorbed radiation energy as heat in the glass matrix what can stimulate thermoluminescence effect.

**Figure 3:**
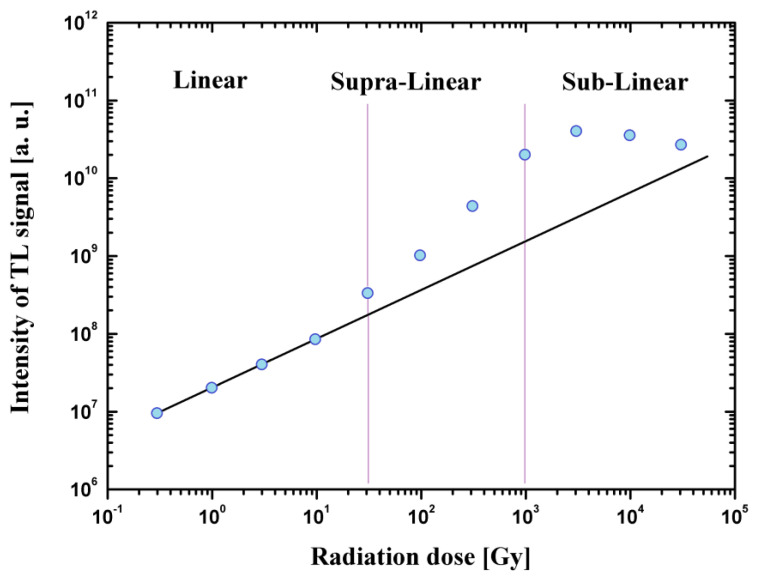
An example of the linear, super-linear and sub-linear thermoluminescence signal as a function of the absorbed dose [124].

For most TL detectors, the dose response is linear to about a few Gy when it becomes super-linear or sublinear until the detector signal is saturated. The TL response as a function of dose can be described by the function:

(1) $$f\left(d\right)=\frac{S\left(d\right)/d}{S\left(D\right)/D}$$

where S(d), S(D) are the indications of the detectors for the dose d and D, respectively, where the dose D is measured in the linear region of detector. In the dose region, where f(d) = 1, the response of the detector is linear with the dose, where f(d) > 1 we have the region of super-linearity and where f(d) < 1 we have sublinear region. Figure 3 shows the linearity, super-linearity and sub-linearity of the exemplary thermoluminescence signal as a function of the absorbed dose.

Phosphate glass doped with 0.5 mol% of lanthanum oxide exhibits 2 luminescence regions in the ranges of blue and red light [125]. The main thermoluminescence peaks appear between 100 and 140 °C for the temperatures up to 300 °C. The deconvolution process of the TL spectrum into Gaussian shape peaks in the range of 100–140 °C reveals two emission energies at 2.01 eV and 2.70 eV (~615 and ~460 nm, respectively). After one day of storage (24 h) in the black box, the initial TL intensity is reduced to 50.8% for the phosphate glass doped with La_2_O_3_. It is worth noting that for a phosphate glass doped with yttrium oxide, the glow curves are characterized by TL peaks in the similar range (100–140 °C) but with lower intensities [126]. These TL spectra comparison indicates that La and Y, in contrast to other rare-earth or transition metal dopants, do not directly act as a recombination center for phosphate glass but possibly enhance luminescence efficiency stabilizing other point defects such as vacancies by the charge compensation mechanism.

The energy transfer processes lead to higher TL yield in Ce^3+^/Tb^3+^ doped phosphate glasses [127]. A new concept of energy transfer, based on nearly resonant energy migration through a rare-earth-ion subsystem in the glass matrix followed by a single-step transfer to an emission centre, was observed. Gadolinium ions Gd^3+^ at a sufficiently high concentration enable effective energy migration in the phosphate glasses matrices followed by single-step energy transfer towards emission centers created by Ce^3+^ and Tb^3+^ doping ions. Moreover, it has been observed that strongly increasing efficiency of Ce^3+^ emission in the Gd-containing Na_67_Gd_30_Ce_3_ sample with respect to Gd-free Na_97_Gd_0_Ce_3_ one. Furthermore, an increase in the TL signal is observed by increasing the concentration of Tb^3+^ ions. On the other hand, a lower signal is detected by increasing the concentration of Gd^3+^. The thermoluminescence measurements above room temperature show the presence of two types of traps with concentrations strongly dependent on the glass composition. Cerium-doped barium borate glasses exhibit both thermoluminescence (TL) and optically stimulated luminescence (OSL) phenomenon [114]. The increase in the cerium dopant concentration from 1 to 5 mol% causes the efficiency enhancement of TL by more than ten times and OSL by more than one hundred times. Moreover, the process of controlled crystallization leads to formation of the optically active BaCeB_9_O_16_ phase and higher TL and OSL signals at lower temperature. The shape of the glow curves confirms the linearity of the recorded TL signal as a function of the increasing radiation dose [128]. Unfortunately, barium borate glasses are not the most chemically resistant among wide variety of glass systems. The main disadvantages include the lack of resistance to the presence of moisture in the air. Currently, attempts are being made to increase the resistance to dissolution of borate glasses by adding, e.g., silica SiO_2_ [129]. As the amount of silica increases, the chemical resistance increases, but at the expense of the amount of optically active phase, which reduces the value of the luminescence signal. Therefore, it is crucial to choose the optimum amount of SiO_2_ to maintain a balance between luminescent properties and thermal stability.

The presence of trivalent cations (dopants) results in a more stable (less hygroscopic) glass structure in all of the glass samples. However, the solubility limit related to the overall concentration of trivalent ions in phosphate glasses seems to be round 50 mol% [130]. The tellurite glasses doped with praseodymium ions Pr^3+^ exhibit reddish-orange luminescence under blue wavelength excitation [131]. This phenomenon suggests its possible application in tri-color white light emitting diodes as a red component. Praseodymium is also used with success in various types of phosphors due to the efficient reddish-orange emission of light [132]. For example, CdGeO_3_ phosphor exhibits long persistent luminescence (LPL) after the short UV-irradiation. The reddish-orange photostimulated luminescence (PSL) is also observed upon near infrared stimulation at 980 nm after per-exposure into UV light. The light emission is caused by the presence of Pr^3+^ ions in trapped states in the structure of the lattice. The most effective concentrations of Pr^3+^ ions for the brightest photoluminescence (PL) emission and the best LPL characteristic are about 3 mol% and 0.5 mol%, respectively. The incorporation of 0.3 mol% Pr_6_O_11_ to germano-tellurite glasses enhances the intensity of the thermoluminescence response, but in different ways for series with and without Ga_2_O_3_ [133]. The inversion of the maxima of the main TL peaks between both series is noticeable. The additive of Ga_2_O_3_ inhibits phase separation of Te-Ge oxide network. The Ga^3+^ ions play a role of the modifier and facilitate connection between structural units of two glass-formers, preventing phase separation.

The zinc phosphate glass doped with Y_2_O_3_:Eu^3+^ powder is characterized by good transparency above 90% for the range 400–700 nm [125]. The bimetallic nanoparticles (BMNPs) of Y_2_O_3_ were synthesized by evaporation method at 1100 °C with 4.3 mol% admixture content of Eu^3+^. The doped glasses exhibit an intense thermoluminescence emission, characteristic for intra-electronic transition energy levels of Eu^3+^ ion. The main luminescence emission wavelength is about 613 nm, which corresponds to the ^5^D_0_ to ^7^F_2_ transition. Meanwhile, the main excitation wavelength which was observed was approximately *λ_exc_* = 395 nm. Thus, it is possible to induce luminescence in these glasses with a standard blue laser (400–410 nm). The chromaticity diagram (CIE) confirmed that synthesized glasses with BMNPs have emission of light in the red range [125].

The addition of Dy_2_O_3_ to borate glasses acts as a network modifier which also exhibits luminescence properties. The small amount of dysprosium oxide can change the coordination number of boron atoms from 3 to 4, which results in greater number of non-bridging oxygens [134]. It provides better efficiency of thermoluminescence processes in the borate glasses. The presence of Dy^3+^ ions in lithium borate network plays a crucial role for the luminescence process due to onefold trapping point behavior [135]. As a result, the probability of the hole-electron recombination process increases because of re-trapping mechanisms are negligibly small. Moreover, the indirect and direct optical bandgap energy of doped lithium borate glass decreases with higher amount of dysprosium ions [136]. Mainly, it is caused by the presence of bonding defects and increasing number of non-bridging oxygens in the glass network. Simultaneously, the incorporation of dysprosium oxide to the glass matrix induces the higher amount of donor energy levels and the average value of Urbach energy increases with greater amount of Dy_2_O_3_ content. This tendency also confirms that dysprosium ions can induce trapping defects in the glass network which results in higher glass disordering. The minimal value of Urbach energy is obtained for lithium borate glass with no dysprosium addition. The lack of interstitial defects in the glass network exhibits in better alignment of the structure [137].

Borate glasses doped with erbium ions Er^3+^ have promising thermoluminescence properties in view of high-dose radiation detectors [123]. This type of glass exhibited a good linearity in the 0.25 kGy to 3 kGy range, with a high sensitivity of 27 thousand counts∙g^−1^∙kGy^−1^ and a minimum detectable dose of 0.2 kGy. After 7 days of storage, the integrated TL intensity of erbium-doped borate glasses reduced by just 4% in comparison with the standard response. However, the reduction was 21% and 50% of its standard value after 15 days and 30 days, respectively. Beyond 30 days, stability was observed as the residual signal remained almost the same even up to 90 days [137]. The erbium rare-earth ion (Er^3+^) has notable emission bands in MIR, NIR, red, green, and violet light. Therefore, erbium ions are often used in phosphate glasses as application in optical fibers, lasers, and emission devices [138,139].

Thermoluminescence measurements for several materials show that there are measurable differences between signals from materials in the powder and bulk forms [137]. For example, powdered calcium fluoride, crystalline calcite, aragonite, and Iceland Spar have altered thermoluminescence signal in comparison with the bulk form. For example, particle size selection of rare-earth oxides can increase thermoluminescence emission in the doped phosphate glasses [118]. Studies have shown that the efficiency of TL is the higher the larger particles of Gd_2_O_3_ or Dy_2_O_3_. The use of 500–1000 µm Gd_2_O_3_ as well as Dy_2_O_3_ particles allows a linear thermoluminescent response to doses up to 0.9 kGy. The application of smaller particles up to 25 µm gradually reduces the luminescence efficiency. Thus, the incorporation of dysprosium or gadolinium ions into the glass matrix affects both the structure of the electron traps energy levels in the glass matrix and TL emission intensity with respect to particles size. The obtained phosphate glasses exhibit good thermoluminescence properties for the high energy beta radiation dose measurement applications [118].

**Figure 4:**
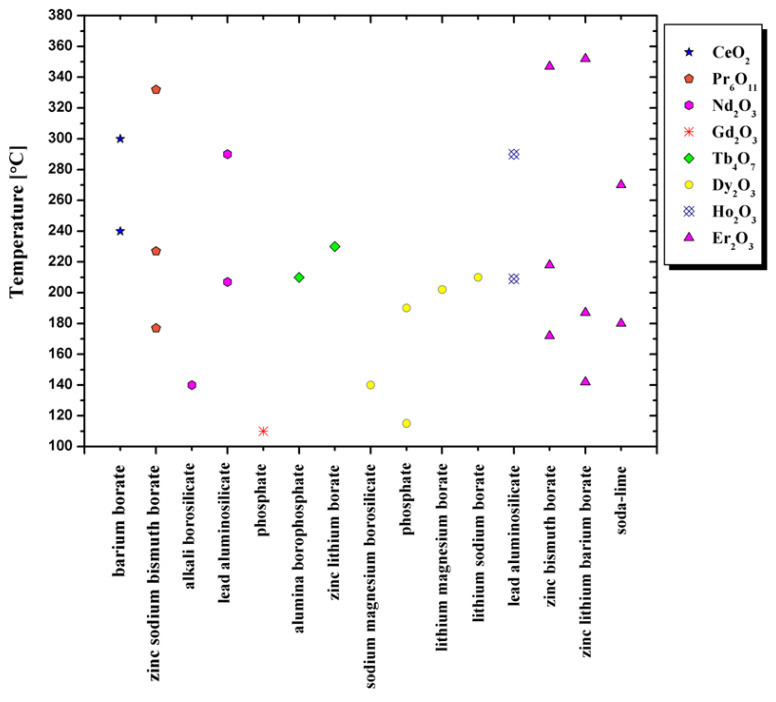
The juxtaposition of the temperatures for the thermoluminescence peak maxima based on various glass systems data [48,114,115,116,117,118,119,120,121,122,123,134,140,141].

The temperature of the TL peak for different glass systems is presented in the Figure 4. The colors of the symbols, with which the appropriate dopants from the lanthanide group are marked, correspond to the luminescence emission colors.

**Figure 5:**
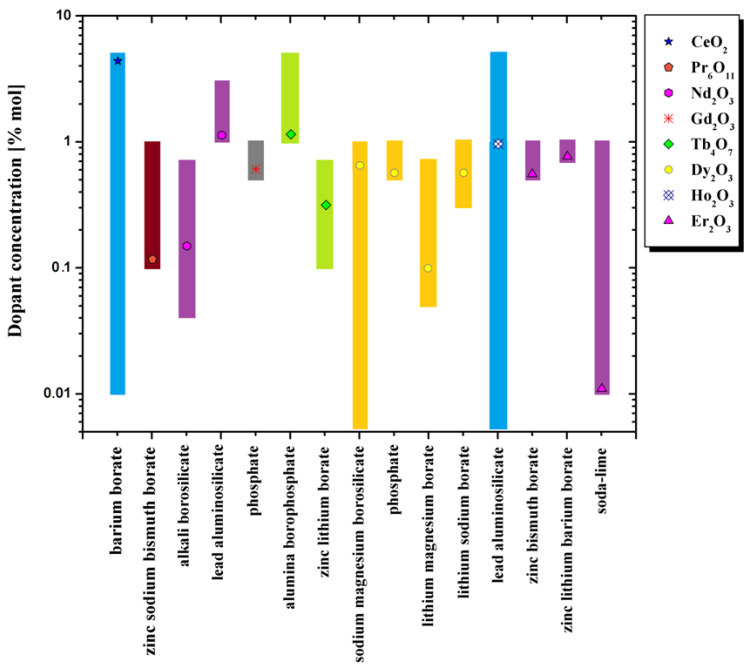
The ranges of rare-earth dopant concentration for various glass systems data. The legend’s markings correspond to the concentration that induces the maximum luminescence signal for a given glass [48,114,115,116,117,118,119,120,121,122,123,134,140,141].

The ranges of rare-earth dopant concentration for various glass systems from Figure 4 are presented in the Figure 5. The legend markings correspond to the concentration that induces the maximum luminescence signal for a given glass.

Fading is a process during which the radiation dose information stored by thermoluminescent detector is lost, mainly due to the thermal influence of external environment. The thermal stability of the stored TL signals under influence of some environmental conditions is specified by fading parameters. Therefore, the developed TL materials are stored in the black boxes at an ambient temperature, while all measurements are taken in the red lighted rooms. It means that only storage time between irradiation and TL readout can influence on the intensity of luminescence response. The characteristic parameters of thermal fading for different glass systems doped with rare-earth elements are presented in Table 2.

**Figure 6:**
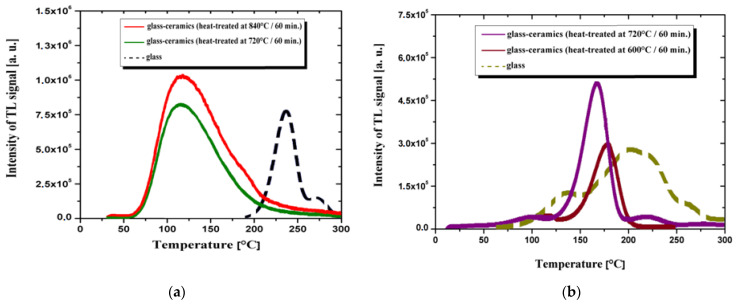
The comparison of the thermoluminescence glow curves for glassy and glass-ceramic material. (**a**) 5CeO_2_-20BaO-75B_2_O_3_ system [114], (**b**) 40LiF-30B_2_O_3_-30SiO_2_ system [51].

As mentioned before, there is a different TL response of the as-made glass and glass-ceramics [51,114]. Figure 6 shows the influence of heat treatment on the intensity of TL signal and temperature of the glow peak. The effect was studied on samples from CeO_2_-BaO-B_2_O_3_ [114] and LiF-B_2_O_3_-SiO_2_ systems [40]. Glass-ceramic materials have been obtained by a heat treatment for 1 h at the temperatures of 720 °C and 840 °C for borate glass, and 600 °C and 720 °C for borosilicate glass, respectively. The significant increase in TL response was observed for glass-ceramics in both cases. Simultaneously, the glow peaks shift to lower temperature. This was caused by crystallization of BaCeB_9_O_16_ and LiF phase, respectively. As an example, the peak intensity of TL signal and temperature of TL peak for the same heating rate were summarized for different kind of materials exposed to 10 Gy in the Table 3. It should be noted that the intensity of the thermoluminescence signal and the shape of the glow curve depend on the sample heating rate.

## 4. Conclusions

The crystalline and amorphous materials were described and compared due to their termoluminescence properties and their application as dosimeters for high energy radiation. This work shows that glassy materials can be as beneficial in dosimetry as commercially used crystalline ceramics. Especially considering lower content of active compounds (i.e., LiF) in the glass, the amorphous materials can provide a similar intensity to the thermoluminescent response. The temperature of the glow peak is generally lower in glassy materials. The chemical resistance of glass to environmental conditions (and even biocompatibility of phosphate glasses) means that they could be used in biological conditions, i.e., in brachytherapy. Furthermore, the glass allows easy fabrication and implementation of rare-earth elements into the more open structure then crystals and in a wider range of concentration, thereby increasing the efficiency of the luminescence. However, it is worth noting that the transformation from glass to glass-ceramics enhance the thermoluminescent properties.

The aim of the review article was to summarize the current knowledge about the thermoluminescent properties of glassy and glass-crystalline materials. Unfortunately, due to the small amount of fading data for glass-ceramic materials, it was not possible to make a complete comparison of glassy and crystalline materials. In addition, the properties of materials whose cross-section for interaction with radiation is similar to that of human tissues dominated the review. It should be noted that there are many other glass systems, such as: silicate, telluride, and germanium, which were only mentioned in the article. Perhaps the above-mentioned shortcomings will be the goal of another review article on the thermoluminescent properties of solids in the near future.

## Figures and Tables

**Table 1:** Linear regions of thermoluminescence dose response for various glass systems.

Glass System	Dopant	Linear Region	References
sodium phosphate	La_2_O_3_	≤33 Gy	[113]
barium borate	CeO_2_	≤10 Gy	[114]
zinc sodium bismuth borate	Pr_6_O_11_	≤3 kGy	[115]
alkali borosilicate	Nd_2_O_3_	≤1 kGy	[116]
lead aluminosilicate	Nd_2_O_3_	≤3 kGy	[117]
phosphate	Gd_2_O_3_	≤10 kGy	[118]
alumina borophosphate	Tb_2_O_3_	≤4 kGy	[119]
sodium magnesium borosilicate	Dy_2_O_3_	≤10 kGy	[120]
phosphate	Dy_2_O_3_	≤10 kGy	[118]
lead aluminosilicate	Ho_2_O_3_	≤5 kGy	[121]
zinc bismuth borate	Er_2_O_3_	≤3 kGy	[122]
zinc lithium barium borate	Er_2_O_3_	≤3 kGy	[123]

**Table 2:** Thermal fading for various glass and glass-ceramics systems.

System	Dopant	Dose	Fading after 7 Days [%]	References
sodium phosphate glass	La_2_O_3_	2 Gy	35	[113]
alkali borosilicate glass	Nd_2_O_3_	10 kGy	22	[116]
zinc lithium borate glass	Tb_4_O_7_	3 Gy	11	[48]
lithium magnesium borate glass	Dy_2_O_3_	5 Gy	22	[134]
lithium sodium borate glass	Dy_2_O_3_	3 Gy	7	[140]
zinc lithium barium borate glass	Er_2_O_3_	3 kGy	4	[123]
soda-lime glass	Er_2_O_3_	14 mGy	45	[141]
crystalline lithium fluoride	Mg,Cu,P	10 mGy	<1	[142]
crystalline lithium fluoride	Mg,Ti	10 mGy	<5	[142]

**Table 3:** TL intensity at the peak and its temperature of various thermoluminescent materials for 10 Gy radiation dose measured with the 5 °C/min. heating rate.

TL Materials	Peak Intensity of TL Signal [×10^6^]	Temperature of TL Peak [°C]
LiF:Mg,Cu,P [38]	2.79	265
Al_2_O_3_:C [40]	1.48	180
CaSO_4_:Mg [41]	1.12	215
LiF:Mg,Ti [38]	0.51	205
CeO_2_-BaO-B_2_O_3_ glass-ceramics (treated at 840 °C) [114]	1.05	120
CeO_2_-BaO-B_2_O_3_ glass-ceramics (treated at 720 °C) [114]	0.78	120
CeO_2_-BaO-B_2_O_3_ glass [114]	0.76	235
LiF-B_2_O_3_-SiO_2_ glass-ceramics (treated at 720 °C) [51]	0.52	160
LiF-B_2_O_3_-SiO_2_ glass-ceramics (treated at 600 °C) [51]	0.29	175
LiF-B_2_O_3_-SiO_2_ glass [51]	0.28	200

## Data Availability

The data that support the findings of this study are available on request from the corresponding author; swiontek@agh.edu.pl.

## References

[1] Noor N.M., Fadzil M.S.A., Ung N., Maah M., Mahdiraji G., Abdul-Rashid H., Bradley D., Abdul-Rashid H. (2016). Radiotherapy dosimetry and the thermoluminescence characteristics of Ge-doped fibres of differing germanium dopant concentration and outer diameter. Radiat. Phys. Chem..

[2] Rivera T. (2012). Thermoluminescence in medical dosimetry. Appl. Radiat. Isot..

[3] Pinto T.N.O., Antonio P.L., Caldas L.V.E. (2011). Measuring TL and OSL of beta radioisotopes inside a glove box at a radiopharmacy laboratory. Radiat. Meas..

[4] Miyahara M.M., Sugi E., Katoh T., Hironiwa T., Sunaga H., Luo L.Z. (2012). Study of effective factors in detection of irradiated food using thermoluminescence based on the models of reference minerals. Radiat. Phys. Chem..

[5] Sears D.W., Sears H., Sehlke A., Hughes S.S. (2018). Induced thermoluminescence as a method for dating recent volcanism. J. Volcanol. Geotherm. Res..

[6] Quickert N.A., Godfrey-Smith D.I., Casey J.L. (2003). Optical and thermoluminescence dating of Middle Stone Age and Kintampo bearingsediments at Birimi, a multi-component archaeological site in Ghana. Quat. Sci. Rev..

[7] Mhareb M.H.A., Alsharhan R., Sayyed M.I. (2021). The impact of TeO_2_ on physical, structural, optical and radiation shielding features for borate glass samples. Optik.

[8] Dwaikat N., Sayyed M.I., Mhareb M.H.A. (2021). Durability, optical and radiation shielding properties for new series of boro-tellurite glass. Optik.

[9] Atri D., Melott A.L. (2014). Cosmic rays and terrestrial life: A brief review. Astropart. Phys..

[10] Niazi A., Khabaz R. (2021). An approach to determination of dosimetric characteristics of radionuclide neutron sources with specific constants and effective quality factors. Radiat. Phys. Chem..

[11] Daneshvar H., Shafaei M., Manouchehri F., Kakaei S., Ziaie F. (2020). Influence of morphology and chemical processes on thermoluminescence response. J. Lumin..

[12] Sugakov V., Ostapenko N., Ostapenko Y., Kerita O., Strelchuk V., Kolomys O. (2017). Molecular vibrations, activation energies of trapped carriers and additional structure in thermoluminescence of organic polymers. Synth. Met..

[13] Cairns T. (1976). Archaeological dating. Anal. Chem..

[14] Topaksu M., Yazici A.N. (2007). The thermoluminescence properties of natural CaF_2_ after β-irradiation. Nucl. Instrum. Methods.

[15] Bos A.J.J. (2007). Theory of thermoluminescence. Radiat. Meas..

[16] Horowitz Y.S. (2019). Thermoluminescence and Thermoluminescent Dosimetry.

[17] McKeever S.W.S. (1988). Thermoluminescence of Solids. Cambridge Solid State Science Serie.

[18] Kron T. (1994). Thermoluminescence dosimetry and its applications in medicine. Phys. Eng. Sci. Med..

[19] Murthy K.V.R. (2013). Thermoluminescence and its Applications: A Review. Defect Diffus. Forum.

[20] Duragkar A., Muley A., Pawar N.R. (2019). Versatility of thermoluminescence materials and radiation dosimetry—A review. Luminescence.

[21] Kadam A.R., Mishra G.C., Dhoble S.J. (2021). Thermoluminescence study and evaluation of trapping parameters CaTiO_3_: RE (RE = Eu^3+^, Dy^3+^) phosphor for TLD applications. J. Mol. Struct..

[22] Delice S., Isik M., Gasanly N.M. (2019). Effect of heating rate on thermoluminescence characteristics of Y_2_O_3_ nanoparticles. J. Lumin..

[23] Bilski P., Obryk B., Gieszczyk W., Baran P. (2020). Position of LiF: Mg, Cu, P TL peak as an alternative method for ultra-high-dose dosimetry. Radiat. Meas..

[24] Kayacan O., Can N. (2004). A generalized method for handling first- and second-order thermal desorption and thermoluminescence. Chem. Phys..

[25] Sholom S., McKeever S.W.S., Chandler J.R. (2020). OSL dosimetry with protective glasses of modern smartphones: A fiber-optic, non-destructive approach. Radiat. Meas..

[26] Bao L., Zha G., Gu Y., Jie W. (2021). Study on radiation damage effects on CdZnTe detectors under 3 MeV and 2.08 GeV Kr ion irradiation. Mater. Sci. Semicond. Process..

[27] Tsu D.V., Schuelke T., Slagter J. (2020). Optical measure of disorder: Why Urbach analysis works for amorphous silicon but fails for amorphous carbon. Diam. Relat. Mater..

[28] Skuja L., Kajihara K., Hirano M., Hosono H. (2012). Oxygen-excess-related point defects in glassy/amorphous SiO_2_ and related materials. Nucl. Instrum. Methods Phys. Res. B.

[29] Elsts E., Rogulis U., Bulindzs K. (2015). Studies of radiation defects in cerium, europium and terbium activated oxyfluoride glasses and glass ceramics. Opt. Mater..

[30] Uklein A.V., Popov A.S., Lisnyak V.V. (2018). Probing of the oxygen-related defects response in Nd: Phosphate glass within self-action of the laser radiation technique. J. Non-Cryst. Solids.

[31] Ma Y., Su H., Zhang Z. (2021). Effect of melting atmospheres on the optical property of radiation-hard fluorophosphate glass. Ceram. Int..

[32] Lu Y., Li P., Xie W. (2021). Negative thermal quenching CsPbBr_3_ glass-ceramic based on intrinsic radiation and vacancy defect co-induced dual-emission. J. Eur. Ceram. Soc..

[33] Hamzelou J. (2011). The Manhattan memory project. New Sci..

[34] Parisi A., de Freitas Nascimento L., Van Hoey O., Mégret P., Kitamura H., Kodaira S., Vanhavere F. (2018). Low temperature thermoluminescence anomaly of LiF: Mg, Cu, P radiation detectors exposed to 1H and 4He ions. Radiat. Meas..

[35] Schwahofer A., Feist H., Georg H., Häring P., Schlegel W. (2017). Experimental determination of the photon-energy dependent dose-to-water response of TLD600 and TLD700 (LiF: Mg, Ti) thermoluminescence detectors. Z. Für Med. Phys..

[36] Iwan A., Bilski P., KŁosowski M. (2010). Thermoluminescence measurements of liquid crystal azomethines and poly(azomethines) with different shapes as thermo-detectors. J. Lumin..

[37] Olko P. (2006). Microdosimetry, track structure and the response of thermoluminescence detectors. Radiat. Meas..

[38] Parisi A., Dabin J., Schoonjans W., Van Hoey O., Mégret P., Vanhavere F. (2019). Photon energy response of LiF:Mg,Ti (MTS) and LiF:Mg,Cu,P (MCP) thermoluminescent detectors: Experimental measurements and microdosimetric modeling. Radiat. Phys. Chem..

[39] Parisi A., Struelens L., Vanhavere F. (2020). The relative efficiency of ^7^LiF:Mg,Ti (MTS-7) and ^7^LiF:Mg,Cu,P (MCP-7) thermoluminescent detectors for muons, pions and kaons over a broad energy range (2 keV–1 GeV): Theoretical calculations using the Microdosimetric d(z) Model. Radiat. Phys. Chem..

[40] Hernández A., Cruz-Zaragoza E., Negrón-Mendoza A., Ramos-Bernal S. (2004). Dependence of thermo-luminescence response of calcium sulphate activated by dysprosium on the temperature irradiation. Radiat. Meas..

[41] Chithambo M.L., Seneza C., Kalita J.M. (2017). Phototransferred thermoluminescence of α-Al_2_O_3_:C: Experimental results and empirical models. Radiat. Meas..

[42] Guckan V., Ozdemir A., Altunal V., Yegingil I. (2019). Studies of blue light induced phototransferred thermoluminescence in CaSO_4_:Mg. Nucl. Instrum. Nucl. Instrum. Methods Phys. Res. B.

[43] Rammah Y.S., Ali A.A., El-Mallawany R., El-Agawany F. (2020). Fabrication, physical, optical characteristics and gamma-ray competence of novel bismo-borate glasses doped with Yb_2_O_3_ rare earth. Phys. B Phys. Condens. Matter.

[44] Qian Q., Zhao C., Yang G., Yang Z., Zhang Q., Jiang Z. (2008). Thermal stability and spectroscopic properties of Er^3+^-doped antimony-borosilicate glasses. Spectrochim. Acta Part A Mol. Biomol. Spectrosc..

[45] Tayal Y., Rao A.S. (2020). Orange color emitting Sm^3+^ ions doped borosilicate glasses for optoelectronic device applications. Opt. Mater..

[46] Pan L., Daguano J.K., Trindade N.M., Cerruti M., Zanotto E.D., Jacobsohn L.G. (2020). Scintillation, luminescence and optical properties of Ce-Doped borosilicate glasses. Opt. Mater..

[47] Hasanuzzaman M., Rafferty A., Sajjia M., Olabi A.G. (2016). Properties of Glass Materials. Ref. Modul. Mater. Sci. Mater. Eng..

[48] Saidu A., Wagiran H., Saeed M., Obayes H., Bala A., Usman F. (2018). Thermoluminescence response of rare earth activated zinc lithium borate glass. Radiat. Phys. Chem..

[49] Zhu D., Ray C.S., Luo F., Zhou W., Day D.E. (2008). Melting and phase-separation of lead borate glasses in low gravity drop shaft. Ceram. Int..

[50] Yadav A., Dahiya M.S., Narwal P., Hooda A., Agarwal A., Khasa S. (2017). Electrical characterization of lithium bismuth borate glasses containing cobalt/vanadium ions. Solid State Ion..

[51] Środa M., Świontek S., Gieszczyk W., Bilski P. (2020). The effect of lithium fluoride on the thermal stability and thermoluminescence properties of borosilicate glass and glass-ceramics. J. Eur. Ceram. Soc..

[52] Puchalska M., Bilski P., Olko P. (2007). Thermoluminescence glow peak parameters for LiF:Mg,Ti with modified activator concentration. Radiat. Meas..

[53] Divina R., Sathiyapriya G., Marimuthu K., Askin A., Sayyed M. (2020). Structural, elastic, optical and γ-ray shielding behavior of Dy^3+^ ions doped heavy metal incorporated borate glasses. J. Non-Cryst. Solids.

[54] Mahmoud K.A., Tashlykov O.L., Sayyed M.I., Kavaz E. (2020). The role of cadmium oxides in the enhancement of radiation shielding capacities for alkali borate glasses. Ceram. Int..

[55] Sadeq M.S., Abdo M.A. (2021). Effect of iron oxide on the structural and optical properties of alumino-borate glasses. Ceram. Int..

[56] Abouhaswa A.S., Sayyed M.I., Mahmoud K.A., Al-Hadeethi Y. (2020). Direct influence of mercury oxide on structural, optical and radiation shielding properties of a new borate glass system. Ceram. Int..

[57] Liu J., Yang K., Zhai J., Shen B. (2018). Effects of crystallization temperature on phase evolution and energy storage properties of BaO-Na_2_O-Nb_2_O_5_-SiO_2_-Al_2_O_3_ glass-ceramics. J. Eur. Ceram. Soc..

[58] Sigel G.H., Ginther R.J. (1968). Effect of iron on ultraviolet absorption of high purity soda-silica glass. Glass Technol..

[59] Duffy J.A. (2001). Ultraviolet transparency of glass: A chemical approach in terms of band theory, polarisability and electronegativity. Phys. Chem. Glasses.

[60] Abdelghany A.M., ElBatal H.A., EzzElDin F.M. (2015). Influence of CuO content on the structure of lithium fluoroborate glasses: Spectral and gamma irradiation studies. Spectrochim. Acta Part A Mol. Biomol. Spectrosc..

[61] Abdelghany A.M., ElBatal H.A. (2016). Optical and μ-FTIR mapping: A new approach for structural evaluation of V_2_O_5_-lithium fluoroborate glasses. Mater. Des..

[62] Schulman J.H., Kirk R.D., West E.J. (1967). Luminescence dosimetry conference. USAEC Symp. Ser..

[63] Takenaga M., Yamamato O., Yamashita T. (1980). Preparation and characteristics of Li_2_B_4_O_7_:Cu phosphor. Nucl. Instrum. Methods.

[64] Sanyal B., Goswami M., Prakasan V., Krishnan M., Ghosh S.K. (2019). Thermoluminescence and electron paramagnetic resonance study on rare earth/transition metal doped lithium borate glasses for dosimetry applications. J. Lumin..

[65] Dhanuskodi S., Mohandoss R., Renganathan B., Sastikumar D. (2014). Transition metal doped nanocrystalline Li_2_B_4_O_7_ for gas sensing applications. Opt. Laser Technol..

[66] Kar S., Debnath C., Verma S., Dhamgaye V.P., Lodha G.S., Bartwal K.S. (2015). Thermoluminescence studies on single crystal, polycrystalline and glass lithium tetraborate samples irradiated by X-rays from Indus-2. Physica B Condens. Matter.

[67] Parauha Y.R., Dhoble S.J. (2020). Thermoluminescence study and evaluation of trapping parameter of rare earth activated Ca_3_Al_2_O_6_: RE (RE = Eu^2+^, Ce^3+^) phosphors. J. Mol. Struct..

[68] Kara U., Issa S.A., Yorgun N.Y., Kilicoglu O., Rashad M., Abuzaid M.M., Kavaz E., Tekin H. (2020). Optical, structural and gamma ray shielding properties of dolomite doped lithium borate glasses for radiation shielding applications. J. Non-Cryst. Solids.

[69] Kashif I., Ratep A., El-Mahy S.K. (2017). Structural and optical properties of lithium tetraborate glasses containing chromium and neodymium oxide. Mater. Res. Bull..

[70] Nikl M., Mares J., Mihokova E., Nitsch K., Solovieva N., Babin V., Krasnikov A., Zazubovich S., Martini M., Vedda A. (2001). Radio- and thermoluminescence and energy transfer processes in Ce^3+^(Tb^3+^)-doped phosphate scintillating glasses. Radiat. Meas..

[71] Timar-Gabor A., Ivascu C., Vasiliniuc Ş., Daraban L., Ardelean I., Cosma C., Cozar O. (2011). Thermoluminescence and optically stimulated luminescence properties of the 0.5P_2_O_5_–xBaO–(0.5x)Li_2_O glass systems. Appl. Radiat. Isot..

[72] Miyazaki A., Kinoshita T., Tatebayashi T., Fang T., Ren Y., Ishiyama T., Nishii J. (2020). Thermal stability and proton conductivity of densely proton injected phosphate glasses containing rare-earth elements. J. Non-Cryst. Solids.

[73] Imaoka M. (1962). Adv. Glass Technology Part I.

[74] Varshneya A.K. (2006). Fundamentals of Inorganic Glasses.

[75] Mackenzie J.D. (1960). Modern Aspects of the Vitrous State.

[76] Koo J., Bae B.S., Na H.K. (1997). Raman spectroscopy of copper phosphate glasses. J. Non-Cryst. Solids.

[77] Ruiz-Aguilar C., Alcántara-Quintana L.E., Aguilar-Reyes E.A., Olivares-Pinto U. (2020). Fabrication, characterization, and in vitro evaluation of β-TCP/ZrO_2_-phosphate-based bioactive glass scaffolds for bone repair. Boletín De La Soc. Es Pañola De Cerámica Y Vidr..

[78] Sekha A.V., Pavić L., Moguš-Milanković A., Purnachand N., Reddy A.S.S., Raju G.N., Veeraiah N. (2020). Dielectric characteristics, dipolar relaxation dynamics and ac conductivity of CuO added lithium sulpho-phosphate glass system. J. Non-Cryst. Solids.

[79] Liu C.-X., Pan H., Lv J.-Y., Chen J.-Y., Lin S.-B., Zheng R.-L., Fu L.-L., Zhang L.-L. (2021). An infrared 2D Nd^3+^-doped phosphate glass waveguide formed by proton implantation and femtosecond laser ablation. Infrared Phys. Technol..

[80] Prasad S., Reddy M.S., Kumar V.R., Veeraiah N. (2007). Specific features of photo and thermoluminescence of Tb^3+^ ions in BaO–M_2_O_3_ (M = Ga, Al, In)–P_2_O_5_ glasses. J. Lumin..

[81] Wahab E.A., El-Maaref A., Shaaban K., Börcsök J., Abdelawwad M. (2021). Lithium cadmium phosphate glasses doped Sm^3+^ as a host material for near-IR laser applications. Opt. Mater..

[82] Murashov A., Sidorov A., Stolyarchuk M., Boiko M. (2017). Effect of X-ray irradiation and thermal treatment on luminescent properties of barium-phosphate glasses doped with silver and copper. J. Non-Cryst. Solids.

[83] Hirano S., Kawano N., Okada G., Kawaguchi N., Yanagida T. (2018). PL and TSL properties of tin-doped zinc sodium phosphate glasses. Radiat. Meas..

[84] Ivascu C., Gabor A.T., Cozar O., Daraban L., Ardelean I. (2011). FT-IR, Raman and thermoluminescence investigation of P_2_O_5_–BaO–Li_2_O glass system. J. Mol. Struct..

[85] Elisa M., Iordache S.-M., Vasiliu I., Grigorescu C., Sava B., Boroica L., Filip A., Dinca M., Bartha C., de Acha N. (2021). Peculiarities of the structural and optical properties of rare-earth-doped phosphate glasses for temperature sensing applications. J. Non-Cryst. Solids.

[86] Manchester R.A., Todorova T.Z., Werner-Zwanziger U. (2021). Mixture designs to investigate the role of alkali and alkaline earth cations on composition-structure-property relationships in ternary borate glass networks. J. Non-Cryst. Solids.

[87] Pugliese D., Veber A., Lemière A. (2021). Effect of post-heat-treatment on the structural, spectroscopic and dissolution properties of a highly stable Er^3+^-doped multi-component phosphate glass. J. Alloys Compd..

[88] Akyildirim H., Kavaz E., El-Agawany F.I. (2020). Radiation shielding features of zirconolite silicate glasses using XCOM and FLUKA simulation code. J. Non-Cryst. Solids.

[89] Kilic G., Ilik E., Issa S.A.M. (2021). Ytterbium (III) oxide reinforced novel TeO_2_–B_2_O_3_–V_2_O_5_ glass system: Synthesis and optical, structural, physical and thermal properties. Ceram. Int..

[90] Gao X., Honma T., Komatsu T. (2021). Enhanced thermal stability and crystallization of nonlinear optical RE_x_Bi_2-x_ZnB_2_O_7_ in RE_2_O_3_-added bismuth zinc borate glasses (RE: Eu, Gd, Er). J. Non-Cryst. Solids.

[91] Shi Q., Yue Y., Qu Y., Liu S., Khater G., Zhang L., Zhao J., Kang J. (2019). Structure and chemical durability of calcium iron phosphate glasses doped with La_2_O_3_ and CeO_2_. J. Non-Cryst. Solids.

[92] Wang E., Li P., Wang L.M. (2018). Strong dependence of the hardness on fictive temperatures in far-from-equilibrium La_57.5_Ni_12.5_Al_17.5_Cu_12.5_ metallic glasses. Intermetallics.

[93] Li H., Yi J., Qin Z., Sun Z., Xu Y., Wang C., Zhao F., Hao Y., Liang X. (2019). Structures, thermal expansion, chemical stability and crystallization behavior of phosphate-based glasses by influence of rare earth. J. Non-Cryst. Solids.

[94] Guedes L.F.N., Marcondes L.M., Evangelista R.O., Batista G., Mendoza V.G., Cassanjes F.C., Poirier G.Y. (2020). Effect of alkaline modifiers on the structural, optical and crystallization properties of niobium germanate glasses and glass-ceramics. Opt. Mater..

[95] Jiménez J.A. (2016). Temperature dependence of Cu^+^ luminescence in barium-phosphate glasses: Effect of rare-earth ions (Sm^3+^, Nd^3+^) and correlation with glass structure. J. Non-Cryst. Solids.

[96] Bardez-Giboire I., Kidari A., Magnin M., Dussossoy J.-L., Peuget S., Caraballo R., Tribet M., Doreau F., Jégou C. (2017). Americium and trivalent Lanthanides incorporation in high-level waste glass-ceramics. J. Nucl. Mater..

[97] Elkholy M.M. (2002). Thermoluminescence for rare-earths doped tellurite glasses. Mater. Chem. Phys..

[98] Abdel-Kader A., El-Mallawany R., Elkholy M., Farag H. (1994). Thermoluminescence dosimetry of rare-earth doped tellurite phosphate glasses. Mater. Chem. Phys..

[99] Bhatia V., Kumar D., Singh H., Kaur N., Rao S., Kumar A., Mehta V., Singh S.P. (2020). Structural, optical and thermoluminescence properties of newly developed MnKB: Er^3+^ glass system. J. Non-Cryst. Solids.

[100] Oliveira M., Galleani G., Magon C.J., Eckert H. (2021). Modern magnetic resonance approaches for characterizing rare-earth containing glasses and glass ceramics. J. Non-Cryst. Solids.

[101] Fu J., Huang Z., Yang J., Ma J., Shen J. (2021). Nano-forming of the rare earth La-based metallic glass. J. Non-Cryst. Solids.

[102] Okura T., Nojima Y., Kawada K., Kojima Y., Yamashita K. (2021). Photoluminescence properties of rare-earth ion-doped Na_5_YSi_4_O_12_-based glass ceramics. Ceram. Int..

[103] Li X., Cao J., Huang M., Peng M. (2021). Modulating broadband near infrared emission from Bi doped borate laser glass by codoping nonactive rare earth ions. J. Non-Cryst. Solids.

[104] Xie W., Fu Q., Cheng C., Yan N. (2020). Experimental and theoretical study on the effect of different rare-earth oxides on the high-temperature stability of SiO_2_ glass at 1973K. Ceram. Int..

[105] Liu L., Chen F., Cui J., Xiao X., Xu Y., Hou C., Cui X., Guo H. (2021). The mutual influence between rare earth element doping and femtosecond laser-induced effects in Ga-As-Sb-S chalcogenide glass. Ceram. Int..

[106] Zhao Y., Bai Y., Ding Y., Hu L. (2020). Predicting the glass-forming ability of rare earth-contained Fe-based alloys by features of dynamic transition in their melts. J. Non-Cryst. Solids.

[107] Enríquez E., Berges C., Fuertes V., Gallego A., Naranjo J., Herranz G., Fernández J. (2020). Ceramic Injection Moulding of engineered glass-ceramics: Boosting the rare-earth free photoluminescence. Ceram. Int..

[108] Marcondes L.M., Rodrigues L., da Cunha C.R., Gonçalves R.R., de Camargo A.S., Cassanjes F.C., Poirier G.Y. (2019). Rare-earth ion doped niobium germanate glasses and glass-ceramics for optical device applications. J. Lumin..

[109] Al-Hadeethi Y., Ahmed M., Al-Heniti S.H., Sayyed M., Rammah Y. (2020). Rare earth Co-Doped tellurite glass ceramics: Potential use in optical and radiation shielding applications. Ceram. Int..

[110] Djamal M., Yuliantini L., Hidayat R., Boonin K., Yasaka P., Kaewkhao J. (2018). Glass medium doped rare earth for sensor material. Mater. Today Proc..

[111] Kichanov S.E., Gorshkova Y.E., Rachkovskaya G.E., Kozlenko D.P., Zakharevich G.B., Savenko B.N. (2019). Structural evolution of luminescence nanoparticles with rare-earth ions in the oxyfluoride glass ceramics. Mater. Chem. Phys..

[112] Ogundare F., Folley D., Chithambo M., Arise T. (2020). Thermoluminescence properties of potassium fluoride. Nuclear Inst. Methods Phys. Res. B.

[113] El-Kheshen A., Woda C., Discher M., El-Faramawy N. (2018). Investigation of phosphate glass doped lanthanum as beta dosimeter. J. Lumin..

[114] Środa M., Świontek S., Gieszczyk W., Bilski P. (2019). The effect of CeO_2_ on the thermal stability, structure and thermoluminescence and optically stimulated luminescence properties of barium borate glass. J. Non-Cryst. Solids.

[115] Hegde V., Viswanath C.D., Chauhan N., Mahato K., Kamath S.D. (2018). Photoluminescence and thermally stimulated luminescence properties of Pr3+- doped zinc sodium bismuth borate glasses. Opt. Mater..

[116] Soliman H.A., Salama E. (2018). Thermoluminescence characteristics and dosimetric parameters of Nd^3+^ doped alkali borosilicate glass. Int. J. Appl. Glass Sci..

[117] Rao M.S., Gandhi Y., Sanyal B., Bhargavi K., Piasecki M., Veeraiah N. (2014). Studies on γ-ray induced structural changes in Nd^3+^ doped lead alumino silicate glasses by means of thermoluminescence for dosimetric applications in high dose ranges. J. Alloys Compd..

[118] Gasiorowski A., Szajerski P. (2020). Particles size increase assisted enhancement of thermoluminescence emission in gadolinium and dysprosium oxide doped phosphate glasses. J. Alloys Compd..

[119] Kalpana T., Sanyal B., Gandhi Y., Kumar V.R., Baskaran G.S., Bragiel P., Piasecki M., Veeraiah N. (2017). Thermoluminescence features of alumina-mixed borophosphate glasses with Tb^3+^ ions for dosimetric applications. Int. J. Appl. Glass Sci..

[120] Kaur R., Bhatia V., Kumar D., Rao S., Singh S.P., Kumar A. (2019). Physical, structural, optical and thermoluminescence behavior of Dy_2_O_3_ doped sodium magnesium borosilicate glasses. Results Phys..

[121] Bhargavi K., Rao M.S., Veeraiah N., Sanyal B., Gandhi Y., Baskaran G.S. (2015). Thermal luminescence of Ho_2_O_3_-PbO-Al_2_O_3_-SiO_2_ glasses exposed to gamma radiation. Int. J. Appl. Glass Sci..

[122] Prabhu N., Hegde V., Sayyed M., Agar O., Kamath S.D. (2019). Investigations on structural and radiation shielding properties of Er^3+^ doped zinc bismuth borate glasses. Mater. Chem. Phys..

[123] Prabhu N.S., Sharmila K., Somashekarappa H.M., Lakshminarayana G., Mandal S., Sayyed M.I., Kamath S.D. (2020). Thermoluminescence features of Er^3+^ doped BaO-ZnO-LiF-B_2_O_3_ glass system for high-dose gamma dosimetry. Ceram. Int..

[124] Farag M.A., Sadek A.M., Shousha H.A. (2017). Radiation damage and sensitization effects on thermoluminescence of LiF:Mg,Ti (TLD-700). Nucl. Instrum. Methods Phys. Res. B.

[125] Mariscal-Becerra L., Carmona-Téllez S., Arredondo-Martínez G., Salas-Mariscal S., Hernández-Sánchez J., Murrieta S H., Falcony C. (2017). Yttrium-europium oxide doped zinc phosphate glasses, a luminescence study. J. Non-Cryst. Solids.

[126] Deepa A.V., Murugasen P., Muralimanohar P. (2018). Optical studies of lanthanum oxide doped phosphate glasses. Optik.

[127] Świontek S., Adamczyk J., Środa M., Bilski P. (2018). Optically active borate glasses doped with cerium in the range of high-energy radiation. Ceram. Mater..

[128] Świontek S., Środa M. (2021). The effect of SiO_2_ on the thermal stability and thermoluminescence properties of barium-cerium borate glass and glass-ceramics. J. Non-Cryst. Solids.

[129] Benyounoussy S., Bih L., El Bouari A. (2020). Influence of niobium oxide content on the structural features of silver phosphate glasses and their corresponding glass-ceramics. Mater. Today Proc..

[130] Trindade C., Alves R., Silva A., Dantas N., Gouveia-Neto A. (2020). Tunable greenish to reddish luminescence and two-way energy transfer in Ho^3+^ and Pr^3+^ doped TeO_2_:ZnO glass. Opt. Mater..

[131] Jin Y., Hu Y., Chen L., Fu Y., Mu Z., Wang T., Lin J. (2014). Photoluminescence, reddish orange long persistent luminescence and photostimulated luminescence properties of praseodymium doped CdGeO_3_ phosphor. J. Alloy. Compd..

[132] Środa M., Świontek S., Fraś D. (2019). Effect of Ga_2_O_3_ on the structure and properties of TeO_2_–GeO_2_ glass doped with Pr^3+^. J. Non-Cryst. Solids.

[133] Ichoja A., Hashim S., Ghoshal S.K., Hashim I.H., Omar R.S. (2018). Physical, structural and optical studies on magnesium borate glasses doped with dysprosium ion. J. Rare Earths.

[134] Hashim S., Omar R.S., Ghoshal S.K. (2019). Realization of dysprosium doped lithium magnesium borate glass based TLD subjected to 1–100 Gy photon beam irradiations. Radiat. Phys. Chem..

[135] Bhogi A., Kistaiah P. (2020). Thermal and structural characterization of lithium borate glasses doped with Fe(III) ions: The role of alkaline earths. Opt. Mater..

[136] Lim T.Y., Wagiran H., Hussin R., Hashim S. (2015). Thermoluminescence response of dysprosium doped strontium tetraborate glasses subjected to electron irradiations. Appl. Radiat. Isot..

[137] Yin L., Townsend P., Wang Y., Khanlary M.R., Yang M. (2020). Comparisons of thermoluminescence signals between crystal and powder samples. Radiat. Meas..

[138] Xiao G., Yan B., Luo Y., Wen J., Fan D., Fu X., Chu Y., Zhang J., Peng G.-D. (2021). Co-doping effect of lead or erbium upon the spectroscopic properties of bismuth doped optical fibres. J. Lumin..

[139] Yusoff N.M., Yao L.K., Sulaiman A.H., Yusoff N.M., Mahdi M.A. (2021). Stable dual-wavelength laser incorporating polarization-maintaining erbium-doped fiber. Opt. Laser Technol..

[140] Alajerami Y.S.M., Mhareb M.H.A., Abushab K., Ramadan K. (2019). Effect of co-doping of lithium on the dosimetric properties of dysprosium-doped sodium borate glass system. Phys. B Condens. Matter.

[141] Laopaiboon R., Thumsa-ard T., Bootjomchai C. (2018). The thermoluminescence properties and determination of trapping parameters of soda lime glass doped with erbium oxide. J. Lumin..

[142] Ptaszkiewicz M. (2007). Long-term fading of LiF:Mg, Cu, P and LiF:Mg, Ti thermoluminescence detectors with standard and modified activator composition. Radiat. Meas..

